# Application of metabolomics and molecular networking in investigating the chemical profile and antitrypanosomal activity of British bluebells (*Hyacinthoides non-scripta*)

**DOI:** 10.1038/s41598-019-38940-w

**Published:** 2019-02-22

**Authors:** Dotsha J. Raheem, Ahmed F. Tawfike, Usama R. Abdelmohsen, RuAngelie Edrada-Ebel, Vera Fitzsimmons-Thoss

**Affiliations:** 1 School of Chemistry, Bangor University, Bangor, Gwynedd, UK; 2 Strathclyde Institute of Pharmacy and Biomedical Sciences, University of Strathclyde, Glasgow, UK; 3 Department of Botany II, Julius-von-Sachs Institute for Biological Sciences, University of Würzburg, Würzburg, Germany; 4 Department of Pharmacognosy, Faculty of Pharmacy, Helwan University, Cairo, 11795 Egypt; 5 Present Address: Department of Chemistry, College of Science, University of Salahaddin, Erbil, Kurdistan Iraq; 6 Present Address: Computational and Analytical Science Department, Rothamsted Research, Harpenden, AL5 2JQ UK; 7 Present Address: Department of Pharmacognosy, Faculty of Pharmacy, Minia University, Minia, 61519 Egypt

## Abstract

Bulb, leaf, scape and flower samples of British bluebells (*Hyacinthoides non-scripta*) were collected regularly for one growth period. Methanolic extracts of freeze-dried and ground samples showed antitrypanosomal activity, giving more than 50% inhibition, for 20 out of 41 samples. High-resolution mass spectrometry was used in the dereplication of the methanolic extracts of the different plant parts. The results revealed differences in the chemical profile with bulb samples being distinctly different from all aerial parts. High molecular weight metabolites were more abundant in the flowers, shoots and leaves compared to smaller molecular weight ones in the bulbs. The anti-trypanosomal activity of the extracts was linked to the accumulation of high molecular weight compounds, which were matched with saponin glycosides, while triterpenoids and steroids occurred in the inactive extracts. Dereplication studies were employed to identify the significant metabolites via chemotaxonomic filtration and considering their previously reported bioactivities. Molecular networking was implemented to look for similarities in fragmentation patterns between the isolated saponin glycoside at *m*/*z* 1445.64 [M + formic-H]^−^ equivalent to C_64_H_104_O_33_ and the putatively found active metabolite at *m*/*z* 1283.58 [M + formic-H]^−^ corresponding to scillanoside L-1. A combination of metabolomics and bioactivity-guided approaches resulted in the isolation of a norlanostane-type saponin glycoside with antitrypanosomal activity of 98.9% inhibition at 20 µM.

## Introduction

*Hyacinthoides non-scripta* (L.) Chouard ex. Rothm., commonly known as British bluebell, are plants native to areas in north-west Europe including the British Isles^1,2^. In the UK, bluebells’ characteristic blue-purple flowers cover wide areas in mid to late spring. Bluebells mostly propagate by seed formed post flowering and are dormant during late summer and autumn. Shoots emerge in mid-winter. Bluebells utilise fructans to support their unusual growth phenology during the colder months in the Northern hemisphere^3^.

Known metabolites of bluebells are the biologically active imino sugars, DMDP ((2*R*, 3*R*, 4*R*, 5*R*)-2, 5-dihydroxymethyl-3, 4-dihydroxy pyrrolidine) and homo-DMDP (2, 5-dideoxy-2, 5-imino-DL-glycero-D-mann-heptitol)^4,5^. Oil has been isolated from the seeds and found to contain a high percentage of monounsaturated fatty acids that includes 20% gondoic acid^6^. During the plant’s flowering season, when the eye catching blue carpets are formed, bluebell flowers yielded mainly delphinidin-3-(6-*p*-coumarylglucoside)-5-(6-malonylglucoside)^7,8^. Other natural products afforded by plants of the family *Hyacinthaceae* have also been reviewed^9^.

Trypanosomiasis is a wide spread disease in sub-Saharan Africa caused by the parasite *Trypanosoma brucei brucei* that affects both humans and animals.The current disease treatment and drugs suffer from limitations due to toxic effects, difficulty in administration, cost and resistance by the parasite^10,11^ hence possible alternative treatments for the disease are sought^12–14^. The combination of biological activity testing of crude extracts with metabolomics accelerates drug discovery, partly because crude extracts show higher biological activity^15^, and partly due to the ability to discriminate between complex mixtures of metabolites^16–22^. Commonly Liquid Chromatography – Mass Spectrometry (LC –MS) and LC – High Resolution (HR) MS is used in metabolomics^23,24^.

LC –HRMS data is processed by differential expression analysis software, such as Mzmine, which involves peak detection, peak deconvolution, isotope grouping, noise removal, and peak alignment to correct deviations in retention time. Dereplication is then performed to identify known metabolites from relevant databases (e.g., Dictionary of Natural Product (DNP) and MarineLit^22,25,26^).

As a metabolomics study yields large amount of data, multivariate data analysis (MVDA) is applied to draw conclusions from the results^27^. MVDA can be applied either as a supervised or as an unsupervised method of analysis. The unsupervised method is exemplified by principal component analysis (PCA) while the supervised method is demonstrated by partial least square (PLS) projection to latent structures (alternatively, orthogonal partial least square projection to latent structures differential analysis (OPLS-DA)). PCA is an effective means of unbiased reduction of dimensionality that reveals groupings or cluster structures where variability between groups is higher than within groups. PLS algorithms allow visualising the separation between groups^28,29^. The application of PCA provides impartial initial conclusions on data variations and similarities, which could be confirmed on PLS plots. PCA and PLS results are presented in score and loading plots. In this paper, the OPLS-DA loading S-plot was used to predict the bioactive metabolites from the anti-trypanosomal active fractions. Targeted isolation work was performed on the predicted bioactive metabolites.

Another approach that aids in the recognition and dereplication of structurally related chemical derivatives is molecular networking. In this study, correlations were based on the MS/MS data of the respective metabolites on the principle that metabolites of similar chemical structures exhibit similar fragmentation patterns under identical ionisation conditions. Molecular networking is complementary to the dereplication approach used in this study. The resultant network of the analysed metabolites is comprised of clusters of nodes with compounds of higher similarity interconnected and often showing relatively high cosine scores^30,31^.

This investigation targeted the chemical metabolites of British bluebells with possible activity against the blood stream form of *Trypanosoma brucei brucei*. MVDA of the occurring secondary metabolites and molecular networking of their HRMS and MS/MS data were utilised as natural product discovery strategies to aid the targeted chromatographic isolation of the bioactive metabolites of the crude plant extracts. Structure elucidation of the isolated compounds was supported by utilising 1D and 2D NMR as well as HRMS data.

## Materials and Methods

### Samples

Bluebell plants used in this study were collected under licence (Grid Reference SH 257 359). Soil and above ground plant material were taken using a stainless steel square hollow section (dimensions 20 cm by 20 cm and 30 cm depth). Bluebell plants were removed from this soil sample and separated into different tissue types (bulbs, leaves, scapes, shoots and flowers). The tissue types were combined, freeze-dried (CHRIST Alpha 1-2 LD plus) and ground using pestle and mortar. Each sample contained approximately 20 individual plants. The first sample was taken in March 2014 of plants with an emerged shoot. The end of the plant’s life occurred in July followed by the subterranean phase (during the end of July- October). On three occasions, samples from the time of shoot emergence and re-growth in the following growth period were also taken to account for one full growth period. Sampled plant parts are given in Table 1.

**Table 1:** Sample codes for bluebell plant parts and collection dates.

Sampling date	Bulbs	Leaves	Scapes	Shoots	Flowers
24 March 2014	1			30	
03 April 2014	2	16	22		34
16 April 2014	3	17	23		35
01 May 2014	4	18	24		36
15 May 2014	5	19	25		37
29 May 2014	6	20	26		38
12 June 2014	7	21	27		39
03 July 2014	8		28		40
17 July 2014	9		29		41
30 July 2014	10				
13 August 2014	11				
29 October 2014	12				
09 February 2015	13			31	
04 March 2015	14			32	
16 March 2015	15			33	

The plants were divided into bulbs, leaves, scapes, shoots and flowers. The shoots refer to the early emergence of the plant when it was not possible to classify the above ground growth into leaves, scapes, and flowers.

Freeze-dried and powdered plant tissues (100 mg) were extracted with MeOH on a 10 mg dry weight to 1 mL of solvent ratio. The samples were shaken at 250 rpm for 30 min. After this period, the samples were centrifuged for 30 min at 5000 rpm, the clear supernatant was separated and the solvent was evaporated. Aliquots of 1 mg of the dried extracts were transferred into LC vials for the subsequent metabolomics profiling analysis.

### Metabolomics Analysis and Profiling

#### Liquid Chromatography – High Resolution Fourier Transform Mass Spectrometry (LC-HRFTMS) Analysis

HPLC analysis was carried out using DionexUltiMate 3000-Thermo Scientific. Dried plant extracts (1 mg) were dissolved in 1 mL HPLC-grade MeOH. The samples were eluted through a C-18 column (ACE, 75 mm, id 3.0 mm, particle size 5 μm). The mobile phase used 0.1% formic acid in HPLC-grade water (solvent A) and acetonitrile (solvent B). The flow rate was 300 µL/min. Gradient elution was employed, commencing at 10% B, held for 5 minutes, increased to 100% B over 30 minutes, held for another 5 minutes before decreasing back to 10% B, held for 4 minutes. Solvent blank refers to sample containing solvent only which was run for the purpose of subtracting background spectra.

Mass spectrometry was carried out using an Exactive mass spectrometer with an electrospray ionization source over a mass range of 100–2000 m/z in positive and negative ionization modes with spray voltage of 4.5 kV and capillary temperature at 270 °C. LC-MS data was acquired using Xcalibur version 2.2.

Data dependent MS2 and MS3 experiments were carried out using a Finnigan LTQ Orbitrap coupled to a Surveyor Plus HPLC pump and autosampler (Thermo Fisher, Bremen, Germany) in positive and negative ionization modes using a mass range of 100–2000 m/z and 30,000 resolution. The capillary temperature was 270 °C, the ion spray voltage was 4.5 kV, the capillary voltage 35 V, the tube lens voltage 110 V and the sheath and auxiliary gas flow rates were 50 and 15, respectively (units not specified by manufacturer).

#### Mass Spectral Data Processing and Metabolomic Profiling

Mass spectral data was processed using the MZmine 2.20 freeware (http://mzmine.sourceforge.net/)^25^. Throughout the data processing, the m/z tolerance used was 0.001, peaks were detected above 5.00E4 and the minimum time span and t_R_ tolerance was 0.2 minutes. Mass detection was performed using centroid mass detector with a noise level set at 1.00E4. The peaks were filtered using FTMS shoulder peaks filter with a mass resolution of 50,000 and the Gaussian peak model.

Chromatograms for each sample and solvent blank were generated to identify the individual peaks and the solvent blank peaks were substracted from the samples. In addition to [M + H]^+^, Na^+^, K^+^ and NH_4_^+^ adducts were searched for after positive mode ESI. In negative mode ESI HCO_2_^−^ adducts were identified, while for both modes CH_3_CN adducts were considered.

Molecular formula prediction was performed for unknown compounds with the number of hits limited by assigning the maximum number of atoms expected of each element (C, H, O, N) along with the mass range of the putative secondary metabolites in the plant extracts. Formula prediction was accomplished using the element count heuristic rules^32^ and double bond equivalence restrictions (RDBE) with the enabled isotope pattern filter. The processed positive and negative raw data were then converted to CSV files and imported into an in-house macro written on Excel^25^. Peaks with intensities 20 times greater in the samples than in the blank solvent were retained. Through EXCEL, macros were also run to match the *m*/*z* peaks with the DNP database version 2015^33^ to enable dereplication of the ion peaks at a *m*/*z* threshold of ±3 ppm, which provided a list of known and unknown metabolites according to their peak intensity. This data was exported to SIMCA V 15.0 (Umetrics, Umeå, Sweden) and further analysed using both PCA and OPLS-DA with Pareto scaling.

### Molecular networking

LC-MS/MS data were subjected to a molecular networking analysis at Global Natural Products Social (GNPS) Molecular Networking website. Metabolite’s networks were then visualized using Cytoscape 2.8 X according to the steps mentioned on their website https://bix-lab.ucsd.edu/display/Public/Molecular+Networking+Documentation.

### Isolation of saponins from bluebell flowers

#### Preparation of the bluebell flower extract

Dried bluebell flowers (124 g) were extracted with MeOH (2.5 L) by soaking overnight and occasional stirring. The extraction was repeated two more times. The extracts were combined and dried down using a rotary evaporator at temperatures below 40 °C. The purple coloured residue was dissolved in water and adsorbed onto 150 mL of HP20 gel previously activated by stirring with MeOH followed by water each for 1 hr. The HP20 gel was separated from the unbound part and washed with deionised water. Then methanol was used to elute the flower extract, which was collected and dried down giving a dark purple-coloured powder (11.13 g).

#### Fractionation of the flower extract

A total of (9 g) of the MeOH extract was fractionated. Automated flash chromatography was carried out using Reveleris Automated Flash System where the chromatographic run was monitored with both evaporative light scattering detector (ELSD) and a UV detector. 18 portions, 500 mg each, of the pigment were individually adsorbed on (2 mL) of silica and dry loaded onto a 12 g Reveleris C18 column. Flash chromatography was carried out applying a flow rate of 18 mL/min and a default fraction volume of 25 mL for all separations. The chromatographic separation was performed using a solvent system of A: MeCN and B: H_2_O both acidified with 0.1% formic acid (FA). The method started with initial 2 min hold at 100% B, then 100–86% B (2 min), 86–80% B (60 min), 80–62% B (11 min) and held at 62% B for 5 min. Combined fractions of 4500 mg were collected at t_R_ 75 min and used for further purification. From this fraction, 300 mg was taken and dissolved in 6 mL de-ionised water and 1 mL portions were injected onto a 12 g Reveleris C18 column. Gradient elution was applied using a solvent system of A: MeCN and B: H_2_O both acidified with 0.1% FA starting from 100% B for 2 min then 100–70% B in 1.5 then 70–62% in 30 min. The purified saponin (20.3 mg) was collected at t_R_ = 11.0 min as a white powder upon solvent evaporation.

#### Spectrometric analysis (MS and NMR) of the isolated saponin

NMR spectra were recorded on a Bruker Avance (400 and 500 MHz) spectrometer. A set of experiments was run including ^1^H, ^13^C, COSY, HSQC, HMBC, HSQC-TOCSY and NOESY. Pyridine-D_5_ (Cambridge Isotope Laboratories, Inc) was used as the NMR solvent and the obtained spectra were referenced using the internal signal from the solvent.

High resolution ESI-MS mass spectra for the isolated compound were obtained using Thermo-Finnigan LTQ Orbitrap (Thermo Scientific, Germany).

### Biological activity

#### Antitrypanosomal Activity

Antitrypanosomal activity of the plant extracts was assessed using 10^4^ trypanosomes per mL of *Trypanosoma brucei brucei* strain TC 221 cultivated in Complete Baltz Medium. Trypanosomes were tested in 96-well plate chambers against different concentrations of test substance in DMSO (0.25–40 µM). The plates were incubated at 37 °C in an atmosphere of 5% CO_2_ for 24 h. After addition of 20 µL of Alamar Blue, viable cells were assessed measuring absorbance at 550 nm using an MR 700 Microplate Reader after 48 and 72 h. The effect of the test compound was quantified in IC_50_ values by linear interpolation of three independent measurements^34^.

The isolated saponin was tested against the bloodstream form of *Trypanosoma brucei brucei* S427. Samples were initially screened at a single concentration of 20 µM (n = 3). The compound was prepared as 10 mM stock solution in 100% DMSO and diluted with HMI-9 (10% FBS) medium. Controls included a sterility control, 0.2% DMSO and a concentration range of suramin as a positive control. Suramin gave an antitrypanosomal activity against *T. b. brucei* at an MIC of 0.11 μM. Trypanosomes were counted using a haemocytometer and diluted to a concentration of 3 × 10^4^ trypanosomes/mL (HMI-9 (10% FBS)), 100 µL of this suspension was added to each well. The assay plate was incubated at 37 °C with a humidified atmosphere containing 5% CO_2_ for 48 hours. Then 20 µL of Alamar blue was added and the incubation continued for a further 24 hours. Fluorescence was then determined using a Wallac Victor 2 microplate reader (Excitation 530 nm Emission 590 nm). The results were calculated as % of the DMSO control values.

## Results and Discussion

### Metabolomics profiling of British bluebell extracts

**Figure 1:**
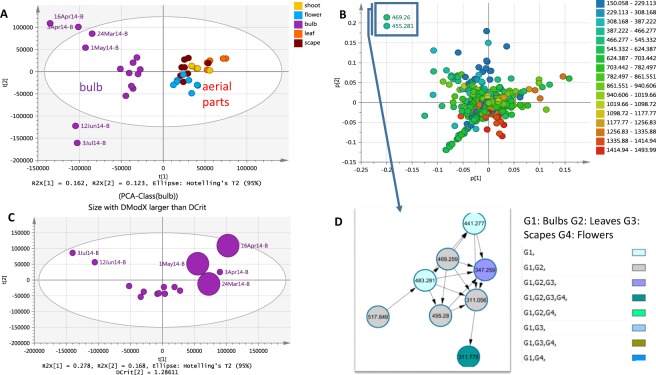
(**A**) PCA scores scatter plot of different plant extracts showing discrimination of bulb extracts, (**B**) PCA loadings plot showing metabolites contributed to the variation, (**C**) PCA-class scores scatter plot of the bulb extracts showing the outlying bulbs due to seasonal variation, (**D**) Molecular network indicating similarity in the fragmentation pattern between the discriminatory metabolites.

To investigate a whole plant bluebell metabolome of one population, plant extracts were prepared from various plant parts collected throughout the growth period and were analysed in both positive and negative ion mode by ESI-HRMS. The PCA scores scatter plot of ESI-HRMS data (Fig. 1A) revealed the discrimination of bulb extracts from the other above ground plant extracts. Although the R^2^ and Q^2^ values for the PCA model were quite low (0.448 and 0.149, respectively), the scores scatter plot indicated a significant variation in the chemical profile of the bulb extracts while clearly separating the underground from the aerial parts. The PCA loadings scatter plot (Fig. 1B) showed the distribution of the metabolites (*m*/*z*) for the respective samples positioned at the same quadrant in the scores plot (Fig. 1A). The metabolites were colour-coded according to their *m*/*z* ranges on the PCA loadings scatter plot (Fig. 1B). Metabolites with relatively lower molecular weights were more abundant in the upper left quadrant representing the bulbs collected between March and May or during springtime. On the other hand, distribution of higher molecular weight metabolites were of higher density on the right quadrants of the PCA loadings plot representing the aerial plant parts that included the leaf, shoot, scape, and flower as shown. The first two principle components of the PCA accounted for a variance of 16% (R^2^X[1] = 0.162) and 12% (R^2^X[2] = 0.123), respectively. The higher variance for PC1 (R^2^X[1] = 0.162) accounted for the variation in chemical profile between the underground and aerial parts (Fig. 1A). However, the PCA plot also exhibited a significant dispersal of bulb extracts particularly between two groups involving the collections from late March to early May and those from June to early July. Their dispersal from the other bulb samples reflected the unique chemical fingerprints of the bulbs during mid-spring and the early summer months, respectively. The significance of the outlying samples was further investigated by generating a PCA-class model for the bulbs. A PCA-class score plot shown in Fig. 1C illustrated that none of the samples were considered strong outliers. A larger sized symbol was used for observation points with DModX values twice as large as the DCrit values, as demonstrated by the samples collected on the 24th of March, 16^th^ of April and 1^st^ of May. These mid-spring extracts were then interpreted as moderate outliers^35^. The DModX is the distance of the respective samples to the X plane of the model while DCrit is the critical distance to the model computed from the F-distribution to attain a 95% confidence interval limit. In simpler terms, at a significant level of 0.95, 95% of the observations should have DModX values less than the DCrit value. Dcrit limit was set at 0.05. In this study, bulb samples collected in mid-spring indicated, “these observations were different from the normal observations with respect to the correlation structure of the variables”^35^. This reflected the distinct chemical profile for those extracts, which coincided with the most active growth phase above ground while the bulb diminished prior to the formation of a new bulb^36^.

**Figure 2:**
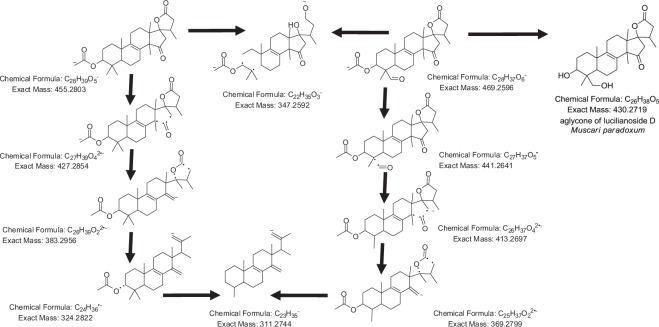
Fragmentation pathway of the acetylated congeners of the aglycone of lucilianoside D found at *m/z* 455.2806 [M − H]^−^ and 469.2597 [M − H]^−^ for C_28_H_40_O_5_ and C_28_H_38_O_6_, respectively.

As shown in the PCA loadings plot (Fig. 1B), searching the compounds that contributed to the variation of the respective discriminant bulb samples revealed that the metabolites at *m*/*z* 455.2806 [M − H]^−^ (t_R_ 31.95 min) and 469.2597 [M − H]^−^ (t_R_ 31.72 min) for C_28_H_40_O_5_ and C_28_H_38_O_6_, respectively, geometrically resembled the quadrant position of the mid-spring extracts presented in the scores plot. Dereplication study of the discriminatory metabolites revealed that the ion peak at *m*/*z* 455.2806 matched 23 plant metabolites from the DNP (Table 2). These metabolites were either triterpenoids or steroids in structures. Furthermore, a molecular correlation network generated from the MS/MS data of the plant extracts (Fig. 1D) revealed the strong structure connectivity between the two metabolites at *m*/*z* 455.2806 and 469.2597 supported by a high correlation cosin value of approximately 0.94. This similarity in fragmentation pattern between the two compounds suggested a comparable structure with only 14 mass unit (mu) differences while the accurate mass established an additional keto substituent with the loss of 2 H and subsequent gain of an oxygen atom. Analysis of the MS/MS data of *m*/*z* 455.2806 and 469.2597 [M − H]^−^ (Fig. 2) suggested the presence of strongly linked metabolites, the structures of which were acetylated congeners of the aglycone of lucilianoside D (Fig. 2) isolated from the fresh bulbs of the Japanese *Muscari paradoxum*^37^. Mass spectral data is presented in the Supplementary Information (Section 1 SI). The fragmentation pattern for both ion peaks involved the subsequent loss of a keto group and CO_2_ that is typical for a five-membered lactone ring system^38^. The loss of CO_2_ in a five-membered lactone ring fragmentation elaborated the simultaneous elimination of H_2_O prior to the loss of a CO unit as observed in both of the spectra for *m*/*z* 455.2806 and 469.2597 [M − H]^−^ (Section 1 SI). The hypothetical structures also exhibited a common mass ion peak fragment at m/z 347.2592 [M − H]^−^ for C_22_H_35_O_3_ as also shown by the molecular correlation network in Fig. 1D, which could be explained by the typical steroid fragmentation pattern^39^ between rings A and B indicating that the 3 oxygen atoms were on ring C or D (Fig. 2).

**Table 2:** Dereplication table of “biomarker” metabolites determined for the different plant parts of British bluebells.

t_R_	*m/z* [ionisation]	MW	Predicted molecular formula/Identification	Earlier reported biological source	Average Intensity
**Group 1. Bulb**					
9.70	542.3326 [M + H]^+^	541.3254	C_28_H_47_NO_9_ picromycin	*Streptomyces venezuelae*	1.39E + 08
31.73	469.2597 [M − H]^−^	470.2670	C_28_H_38_O_6_	*No match*	2.949E + 08
31.95	455.2806 [M − H]^−^	456.2879	C_28_H_40_O_5_ (23 hits) terpenoids steroids	*Solanum cilistum* *Withania somnifera* *Acnistus breviflorus* *Paeonia rockii* *Turraea robusta* *Datura*	2.74E + 08
35.77	483.2751 [M − H]^−^	484.2824	C_29_H_40_O_6_ (19 hits) triterpenoids diterpenoids	*Euphorbia micractina* *Vernonia guineensis* *Caesalpinia echinata* *Dipsacus chinensis* *Datura fastuosa* *Kadsura ananosma* *Maytenus blepharode* *Diospyros decandra*	8.33E + 07
**Group 2. Leaf and Shoot**					
7.22	563.1411 [M − H]^−^	564.1484	C_26_H_28_O_14_ (90 hits) flavonoid glycosides anthraquinone glycosides	widely distributed	2.83E + 08
7.35	563.1411 [M − H]^−^	564.1484	C_26_H_28_O_14_ (90 hits) flavonoid glycosides anthraquinone glycosides	widely distributed	3.86E + 08
8.87	869.2363 [M − H]^−^	870.2436	C_38_H_46_O_23_ 9-(2,3,4,5,6,7-hexahydroxyheptyl) ester, 9′-(shikim-5-yl) ester, 4-O-β-D-glucopyranoside	*Lepicolea ochroleuca*	1.18E + 08
15.64	1269.5766 [M − H]^−^	1270.5838	C_51_H_98_O_35_ C_58_H_94_O_30_	*No match* *No match*	2.63E + 08
15.73	1283.5914 [M − H]^−^ [M + formate-H]^−^	1284.5986 1237.5866	C_59_H_96_O_30_ (Saponins) zingiberenin G platycoside G2 C_58_H_94_O_28_ scillanoside L1	*Platycodon grandiflorum* *Dioscorea zingiberensis* *Scilla scilloides*	7.69E + 08
30.98	695.4662 [M − H]^−^	696.4736	C_39_H_69_O_8_P glycerol 1,2-dialkanoate 3-phosphates C_46_H_64_O_5_	*No match*	2.68E + 08
**Group 3A. Flower and Scape**					
15.20	1445.6454 [M − H]^−^ [M + formate-H]^−^	1446.6526 1400.6444	C_72_H_102_O_30_ C_65_H_106_O_35_ C_58_H_110_O_40_ C_64_H_104_O_33_	*No match* *No match* *No match* *See below*	1.06E + 08
15.24a	1445.6453 [M − H]^−^ [M + formate-H]^−^	1446.6526 1400.6444	C_72_H_102_O_30_ C_65_H_106_O_35_ C_58_H_110_O_40_ C_64_H_104_O_33_	*No match* *No match* *No match* *See below*	1.39E + 08
15.24a	1399.6371 [M − H]^−^	1400.6444	C_64_H_104_O_33_ (Saponins) arganine A*	*Argania spinosa* *Chionodoxa luciliae* *Platycodon grandiflorum* *Mimusops laurifolia* *Achras sapota*	1.21E + 08
15.25a	1401.6545 [M + H]^+^	1400.6473	C_64_H_104_O_33_ (Saponins) arganine A*	*Argania spinosa* *Chionodoxa luciliae* *Platycodon grandiflorum* *Mimusops laurifolia* *Achras sapota*	6.29E + 06
15.55	1415.6346 [M − H]^−^	1416.6419	C_64_H_104_O_34_ (Saponins)	*Platycodon grandiflorum*	1.14E + 08
15.56	1369.6300 [M − H]^−^	1370.6342	C_63_H_102_O_32_ (Saponins)	*Heteropappus altaicus Heteropappus biennis* *Argania spinosa (argan)* *Platycodon grandiflorum* *Crossopteryx febrifuga* *Mimusops laurifolia* *Sideroxylon foetidissimum ssp. gaumeri*	9.24E + 07
15.57	1371.6400 [M − H]^−^	1370.6361	C_63_H_102_O_32_ (Saponins)	*Heteropappus altaicus Heteropappus biennis* *Argania spinosa (argan)* *Platycodon grandiflorum* *Crossopteryx febrifuga* *Mimusops laurifolia* *Sideroxylon foetidissimum ssp. gaumeri*	4.51E + 07

*Dereplicated metabolite is different from that of the isolated compound when their NMR spectral data were compared.

a denotes targeted isolated compound.

**Figure 3:**
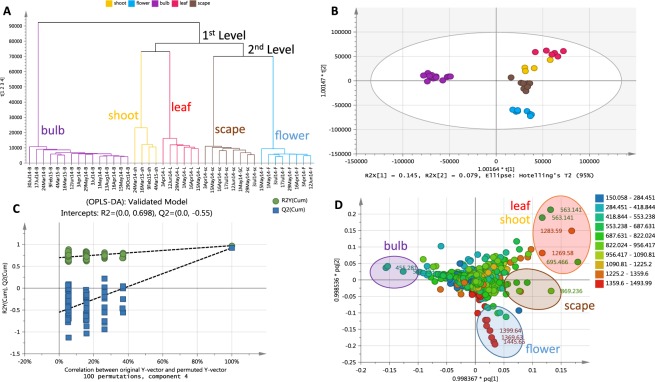
(**A**) HCA plot generated from the OPLS-DA model and (**B**) OPLS-DA scores scatter plot of extracts obtained from different plant parts of British bluebells (R^2^ = 0.948, Q^2^ = 0.748). (**C**) Permutation test plot to validate the OPLS-DA model. (**D**) OPLS-DA loadings scatter plot differentiating the unique metabolites for each of the group cluster as highlighted.

**Figure 4:**
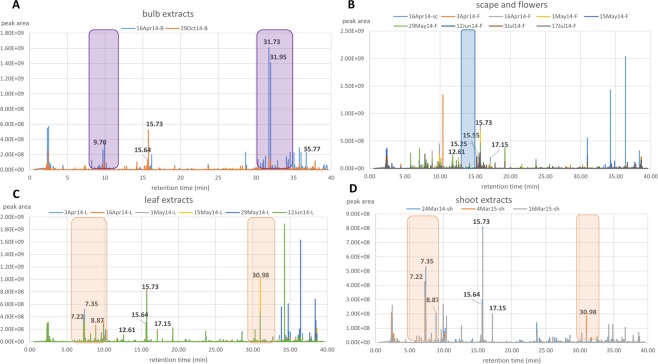
Chromatograms of bioactive crude extracts from respective plant parts: (**A**) bulb, (**B**) scape and flowers, (**C**) leaves, and (**D**) shoots. Highlighted peaks labelled with their retention time represent the “biomarker” metabolites determined for the respective plant parts of British bluebells as shown in Table 2. The other labelled peaks were those found to be the common metabolites in the bioactive extracts.

For a better observation of the chemical variation between the plant parts especially between the various aerial parts, a hierarchical cluster analysis (HCA) (Fig. 3A) was generated from the OPLS-DA scores plot model (R^2^ = 0.948, Q^2^ = 0.748; Fig. 3B). The model was validated by a permutation test (Fig. 3C). The Y intercept (Q^2^Y) on the permutation graph is a measure to check against overfitting. A clear indication that the model is valid and does not happen by coincidence is when the Q2 values of the permuted Y models are less than zero on the permutation plot test^35^. For this study, the model attained a Q^2^Y value of −0.55. Moreover, the difference between Q^2^ and R^2^Y was 0.2, which is less than 0.3, indicating the absence of overfitting. For the HCA dendrogram the clusters were sorted from right to left on the horizontal axis according to increasing observation indices. While the vertical axis indicates the cluster similarity, the variance increases when clusters are merged. As shown by the HCA plot (Fig. 3A), samples were grouped into three main clusters divided into two levels. The first level of the dendrogram established the separation of the underground parts from the aerial parts. The second clustering represented by the aerial parts instigated the second level yielding two groups. The shoot and leaf parts were grouped together while the scape and the flower parts came under one group demonstrating similarities in chemical profiles of these plant parts in each respective group. The scape being a form of a long leafless flowering stem explicated its clustering with the flower parts while on the other hand a shoot system would botanically consist of the stems and leaves. The unique “biomarker” metabolites for the three main clusters have been highlighted on the OPLS-DA loadings scatter plot (Fig. 3D) and listed on Table 2 as also indicated on the chromatograms of the bioactive extracts from their respective plant parts (Fig. 4). The high molecular weight compounds with *m*/*z* [M − H]^−^ range of 1359 to 1500 appear to be concentrated in the flowering parts while the leaf and stem parts yielded metabolites with *m*/*z* [M − H]^−^ ranges of 553 to 822 and 1227 to 1359. From the dereplication study, it can be established that the spirocyclic furanoid nortriterpenes saponins having up to six (6) sugar units were converging in the flower parts while tetranortriterpene spirolactone related saponin aglycones (*m*/*z* 400 to 500 [M − H]^−^) were being accumulated in the bulbs as also observed from the PCA model. On the other hand, the shoot and leaf parts seem to also store saponins but bearing a lower number of sugar units. MVDA has been employed to pinpoint the bioactive metabolites in the crude extracts to guide isolation and purification attempts. Biological investigation of plant extracts for their anti-trypanosomal activity against the blood stream form of *T. brucei brucei* TC221 revealed active and inactive samples. Table 3 presented the IC_50_ values in μg/mL for the tested plant extracts that showed significant inhibition. Biologically active extracts were obtained from the leaves, shoots (except those from the February collection, which was not shown in Table 3), and most of the flowers (excluding the collection from the 15^th^ of May), two bulb samples collected in mid-April and late October and one scape extract from the mid-April collection. Flower samples demonstrated the highest percentage of growth inhibition i.e. flower samples collected at 29^th^ May exhibited 99.1% of growth inhibition with an IC_50_ concentration of 11.08 μg/mL.

**Table 3:** *In vitro* antitrypanosomal activity and IC_50_ values of biologically active methanolic extracts of different parts of bluebell plants against the blood stream form *Trypanosoma brucei brucei* TC221.

Sample code	Sample collection date	Plant part	IC_50_ (µg/mL)	% Growth inhibition
3	16 April 2014	Bulbs	**15.2**	**99.6**
12	29 October 2014	Bulbs	**37.3**	**62.0**
16	03 April 2014	Leaves	**23.0**	**99.5**
17	16 April 2014	Leaves	**22.4**	**99.7**
18	01 May 2014	Leaves	**13.3**	**99.4**
19	15 May 2014	Leaves	**15.8**	**99.9**
20	29 May 2014	Leaves	**23.5**	**99.9**
21	12 June 2014	Leaves	**22.6**	**99.2**
23	16 April 2014	Scapes	**35.8**	**72.0**
30	24 March 2014	Shoots	**22.1**	**99.5**
32	04 March 2015	Shoots	**23.3**	**99.5**
33	16 March 2015	Shoots	**23.5**	**98.6**
34	03 April 2014	Flowers	**14.9**	**99.8**
35	16 April 2014	Flowers	**14.5**	**98.2**
36	01 May 2014	Flowers	**23.5**	**99.2**
37	15 May 2014	Flowers	**40.9**	**57.4**
38	29 May 2014	Flowers	**11.1**	**99.1**
39	12 June 2014	Flowers	**23.3**	**98.4**
40	03 July 2014	Flowers	**18.5**	**99.0**
41	17 July 2014	Flowers	**23.4**	**99.2**

Test solution concentration used is 10 µg/mL and end results were collected after 72 h.

**Figure 5:**
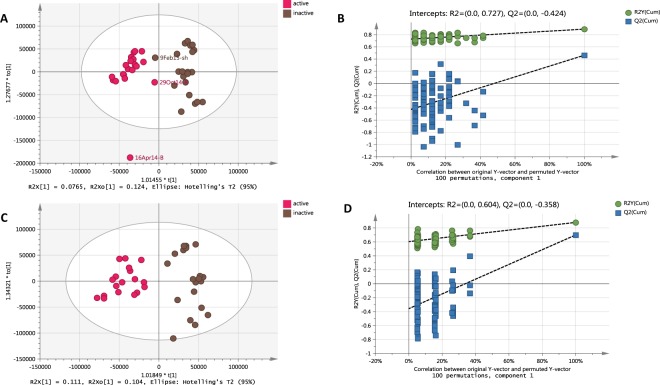
(**A**) OPLS-DA of active versus inactive plant extracts before removing strong outliers, (**B**) permutation test plot for 4A; (**C**) OPLS-DA of active versus inactive plant extracts after removing strong outliers (**D**) permutation test plot for 4C.

In order to pinpoint the structures that were likely to mediate the bioactivity, OPLS-DA was performed on active versus inactive extracts. The model gave good fitness with a R^2^ of 0.891 but gave a low Q^2^ predictability of 0.463. The initial OPLS-DA model (Fig. 4A) gave a low R^2^X[1] variation of 0.0765, which indicated low separation between the active and inactive groups. This was due to the inactive shoot sample collected in early February, which was found on the active left quadrant of the scores scatter plot aligning with the bulb samples collected on 29^th^ of October (Fig. 5A). The bulb extracts obtained from the collection at the end of October gave a weak bioactivity with an IC_50_ value of 37.3 µg/mL, while it inhibited cell growth at only 62.0% at a concentration of 10 µg/mL. This weak activity of the extract was reflected by its position on the OPLS-DA loadings plot. Furthermore, the presence of a strong outlier represented by a bulb sample collected in mid-April could have also affected the Q^2^ score. The within class or common internode variation R^2^X[o2] was at 0.124, which is greater than R^2^X[1] could be due to the seasonal variation resulting to differences in chemical profile of the various plant parts. The model was also validated by a permutation test (Fig. 5B). For this study, the model attained a Q^2^Y value of −0.424, which is an indication that the model is valid. However, the difference between Q2 and R2Y was 0.428, which is greater than 0.3, which may indicate a slightly overfitted model or could have been due to the large variation within the respective groups.

In order to improve the model, the outlier (16 Apr–bulb) and between quadrants samples (9 Feb–shoot and 29 Oct–bulb) were removed, which significantly improved the Q^2^ to 0.699 as shown in Fig. 5C. However, the goodness of fit (R^2^) did not significantly changed at 0.879. This enhanced the separation between the active and inactive samples increasing the R^2^X[1] variation to 0.111 while the common internode variation R^2^X[o2] was also lesser at 0.104. For the permutation test (Fig. 5D), the model attained a Q^2^Y value of −0.358, while the difference between Q^2^ and R^2^Y was decreased to 0.18 which is now less than 0.3 indicating that the model was not overfitted.

**Figure 6:**
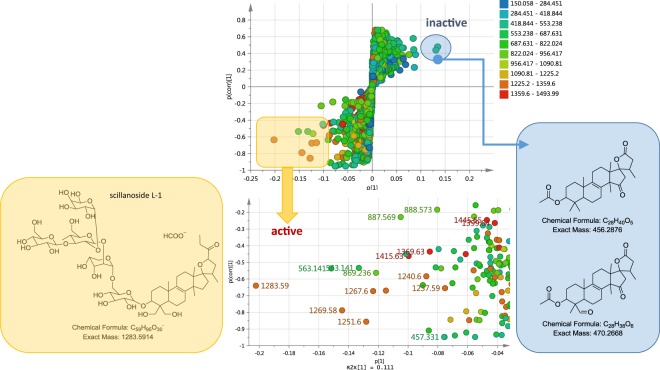
S-loadings plot of active vs. inactive extracts showing the highly correlated putatively active metabolites.

The S-loadings plot (Fig. 6) highlighted the metabolites that highly correlated to the anti-trypanosomal activity of the active extracts. A list of the putatively identified active metabolites is shown in Table 4. Hits were reduced by applying a taxonomic filter and by noting the previously reported biological activities. Metabolites in Table 4 were dereplicated against DNP database and ranked according to their p-value (probability of being responsible for the activity). On top of the list is the ion peak at *m*/*z* 1225.58619 [M + H]^+^ eluting at 15.63 min with a predicted molecular formula of C_57_H_92_O_28_ and was matched to scillascilloside E2^40^. In addition, the compound that possessed the highest covariance is the ion peak at *m*/*z* 1283.5914 (15.73 min) that was a formate adduct of 1237.5866 [M−H]^−^ equivalent to C_58_H_94_O_28_, and matched to scillanoside L-1^40^. Scillanoside L-1 was isolated together with scillascilloside E2 from fresh bulbs of *Scilla scilloides* and both were reported to exhibit variable cytotoxicity against eight cancer cell lines i.e. HT1080 (fibrosarcoma), B16 (F-10) (melanoma), 3LL (lung carcinoma), MCF7 (breast cancer), PC-3 (prostate cancer), HT29 (colon adenocarcinoma), LOX-IMVI (melanoma) and A549 (lung carcinoma) in comparison with adriamycin as a positive control^41^. The rest of the putatively active metabolites in the dereplication table corresponded to previously reported saponin glycosides with various biological activities.

**Table 4:** Dereplication table of the significant and highly correlated putatively active metabolites arranged according to their P-values.

*m/z*	Rt	Identification/molecular formula	Earlier reported biological source	Reported activity (ref.)	Probability P ≤ 0.05
1225.5862 [M + H]^+^	15.63	scillascilloside E2 C_57_H_92_O_28_	*Scilla scilloides*	cytotoxic^41^	3.84E-11
457.3313 [M − H]^−^	15.65	3-dehydro-15-deoxoeucosterol C_29_H_44_O_4_	*Scilla scilloides*	antiinflammatory^44^	3.33E-11
1093.5438 [M + H]^+^	15.63	mycaloside F deapioplatycodin D C_52_H_84_O_24_	*Mycale laxissima* *Platycodon grandiflorum*	antiviral^47^	1.61E-09
1269.5766 [M − H]^−^	15.64	C_51_H_98_O_35_ C_58_H_94_O_30_	No match		8.61E-09
1251.6031 [M − H]^−^	12.61	ardisicrenoside C cauloside H C_59_H_96_O_28_ cf. with scillanoside L-2 C_59_H_94_O_28_	*Ardisia crenata Caulophyllum thalictroides* *Scilla scilloides*	cytotoxic^48,49^	8.43E-08
1267.5977 [M − H]^−^	17.15	soyasaponin A1 C_59_H_96_O_29_	*Impatiens siculifer*	antiinflamatory^50^	1.51E-05
1283.5914 [M + formate-H]^−^	15.73	zingiberenin G platycoside G2 scillanoside L1 C_59_H_96_O_30_	*Platycodon grandiflorum* *Dioscorea zingiberensis* *Scilla scilloides*	cytotoxic^41^	4.51E-04
1237.5866 [M − H]^−^	15.73	scillanoside L-1 C_58_H_94_O_28_	*Scilla scilloides*	cytotoxic^41^	6.27E-04
1239.6016 [M+H]^+^	15.74	scillanoside L-1 C_58_H_94_O_28_	*Scilla scilloides*	cytotoxic^41^	5.02E-04
563.1411 [M − H]^−^	7.35	flavonoid glycosides anthraquinone glycosides C_26_H_28_O_14_	*widely distributed*	cytotoxic^51^	6.47E-04
1415.6346 [M − H]^−^	15.55	platycoside G1 C_64_H_104_O_34_	*Platycodon grandiflorum*	weak cytotoxic^52^	1.40E-02
1369.6269 [M − H]^−^	15.56	heteropappussaponin 7 polygalacin D2 C_63_H_102_O_32_	*Heteropappus altaicus Platycodon grandiflorum*	antiproliferative^53^	1.93E-02

**Figure 7:**
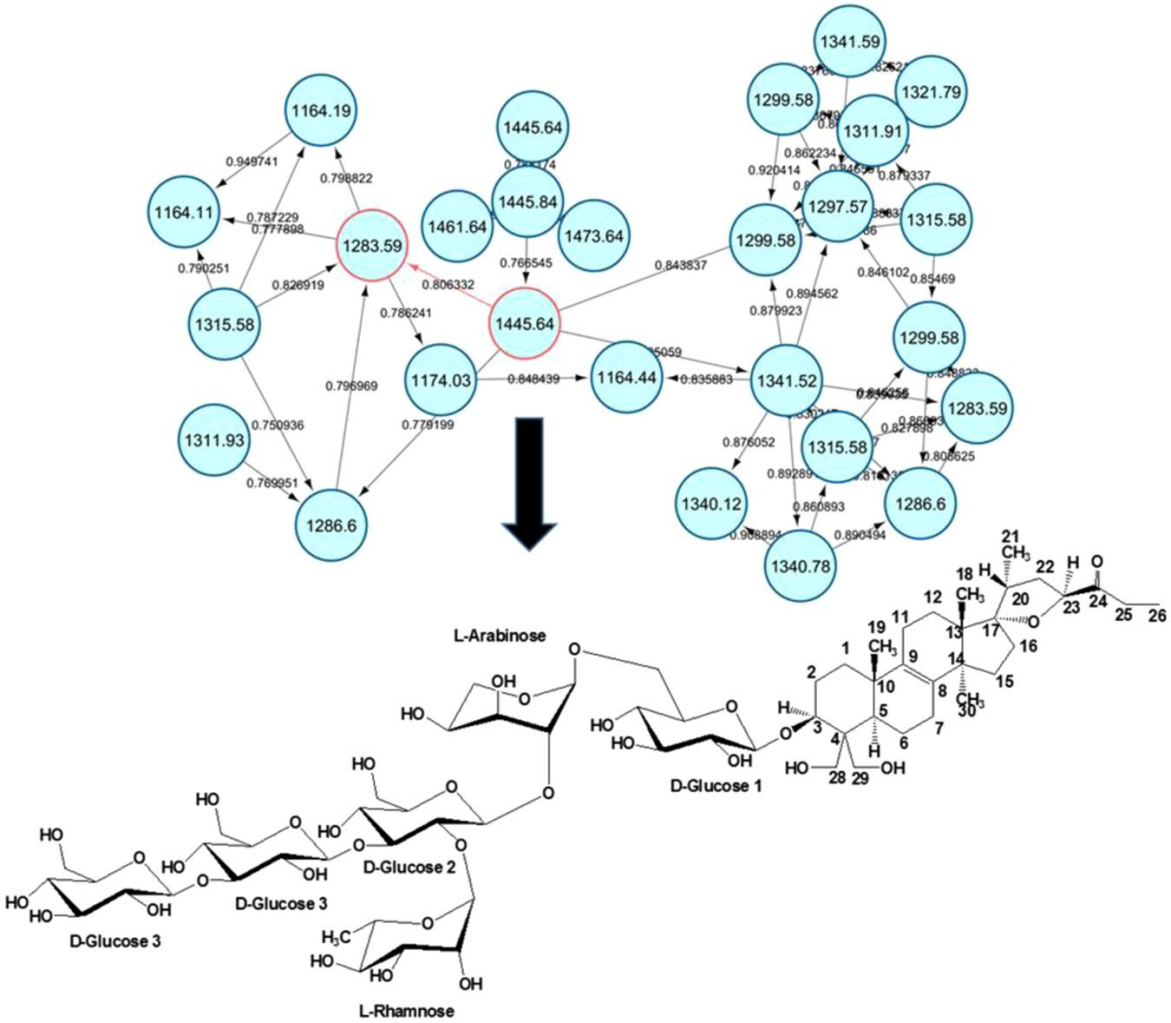
Molecular interaction network showing the neighbour ions of the isolated saponin glycoside *m/z* 1445.645 [M + formate-H]^−^.

The dereplication results encouraged isolation work of the metabolites from the bioactive extracts to explore the putatively active unidentified structures, which were found to be abundant in the flower and leaf extracts, and test purified compounds against *T. brucei brucei*. Due to the relatively higher percentage of flower samples found active against *T. brucei brucei*, a flower extract was chosen for the further isolation work. Bioactivity-guided isolation afforded the potent anti-trypanosomal active saponin glycoside at *m*/*z* 1445.645 [M+formate-H]^−^ which eluted at 15.25 minutes. The compound was designated as ‘unreported’ in the dereplication step as no match in either DNP or the literature was found. As part of the structure dereplication process, a molecular interaction network (MN) of MS/MS data was established to find the nearest correlated structure comparable to this unknown compound. Figure 6 showed the MN generated from the ions of *m*/*z* 1445.645. The MN displayed a strong similarity in the fragmentation pattern (correlation value > 0.80) of *m*/*z* 1445.645 [M + formate-H]^−^ equivalent to C_64_H_104_O_33_ and *m*/*z* 1283.591 (15.73 min) [M + formate-H]^−^ for C_58_H_94_O_28_ corresponding to scillanoside L-1. The mass difference between the two molecules is 162 Da, which is compatible to one sugar molecule [hexose−OH]. The structure (Fig. 7) was supported by HR-MS, MS/MS and 2D-NMR as detailed in the Supplementary Information (Section 2) and is suggested as 3β-(O-β-D-glucopyranosyl-(1 → 3)-O- β-D-glucopyranosyl-(1 → 3)-[α-L-rhamnopyranosyl-(1 → 2)]-O-β-D-glucopyranosyl-(1 → 2)-O-α-L-arabinopyranosyl-(1 → 6)-O-β-D-glucopyranosyl)oxy-17,23-epoxy-28,29-dihydroxy-27-norlanost-8-en-24-one. This saponin exhibited bioactivity when tested against blood stream *Trypanosoma brucei brucei* at a concentration of 20 μM corresponding to 28 μg/mL resulting in 98.9% inhibition. For the extracts showing similar inhibition between 98.2 to 99.9% and having IC_50_ values between 11.1 to 23.5 μg/mL, the isolated saponin was slightly less active, suggesting synergistic effects. Iminosugars could have contributed to these as previously described for the maintenance of sapogenins in the rumen via glycosidase inhibition. Similarly that study found glucosylated saponins more active compared to the aglycones^42^.

A network was also found interconnecting the aglycones and showing a very strong relationship (cosine >0.9), which confirmed a similar fragmentation pattern as well as core basic structure between the respective molecules^30^. Although none of the aglycones were isolated, it was possible to predict their structure using molecular formula prediction tools from Mzmine, which employs the seven golden rules for heuristic filtering of molecular formulas obtained by accurate mass spectrometry^32,43^ supported by their MS/MS fragmentation data and by comparison with possible hits from the DNP^25^. Figure S2 shows the predicted molecular structures for one of these aglycones. More detail is given in section 1 of the SI. The proposed structures were backed by the presence of close structurally related compounds in previous reports from the *Hyacinthaceae*^9,37,44–46^.

This study highlighted the dereplication of saponins using high resolution mass spectral data supported by their fragmentation pattern. The putatively identified congeners were statistically predicted for their bioactivity according to their presence in the active fractions and absence in the inactive fractions. This procedure is not dependent on the concentration of the individual metabolites in the respective fractions, which means a very potent metabolite at very low concentration can be designated as a bioactive metabolite. Some of these putatively identified bioactive metabolites can be present at ug or ng concentrations that are not possible to isolate them with low amount of starting material available.

Only one compound was targeted to be isolated from this work on the basis of its bioactivity and yield or its feasibility to be isolated from the available crude extract. As already mentioned above, the targeted compound did not give any “known hit” from the DNP database and was considered to be a new congener. The elucidation work is presented in the Supplementary Information. Statistically at P < 0.05 (as shown on Table 4), metabolites were predicted to be more potent than the isolated compound (P value at 0.25), one of these compounds is structurally related to scillanoside L-1 with one sugar unit less than the isolated compound. However, as again mentioned above, some of these putatively identified bioactive metabolites can be present at ug or ng concentrations that are not possible to isolate them with very low amount of starting material available. From the S-plot, it was indicated that the aglycones on their own were found to be inactive while their glycosidic counterpart were found more in the active end of the S-plot.

The other compounds described in this study were putatively identified but confirmed from their high resolution mass spectral and fragmentation data while we present in this work the hypothetical fragmentation pathway of the acetylated congeners of the aglycone of lucilianoside D found at *m*/*z* 455.281 [M − H]^−^ and 469.259 [M − H]^−^ for C_28_H_40_O_5_ and C_28_H_38_O_6_, respectively on Fig. 2. The MS-MS fragmentation pathway for the metabolites at *m*/*z* 469.26 and 455.28 were taken in the negative mode. Suggested secondary and tertiary fragmentations are shown in Fig. S3. The high resolution data presented in this study for the dereplicated molecular formula had an average difference of 3 ppm between calculated and found mass.

In conclusion, a combination of metabolomics and bioactivity guided isolation approaches gave access to a shorter route to explore a bioactive saponin glycoside from the British Bluebell, which was simplified due to the implementation of MN for MS/MS data.

## Supplementary information

Supplemenary Information

## Data Availability

The data obtained in this study has been deposited with the European Molecular Biology Laboratory – European Bioinformatics Institute (EMBL-BEI) under study number MTBLS797 and is accessible at https://www.ebi.ac.uk/metabolights/MTBLS797.

## References

[1] Blackman GE, Rutter AJ (1954). Endymion Nonscriptus (L.) Garcke. J. Ecol..

[2] Grundmann M (2010). Phylogeny and taxonomy of the bluebell genus Hyacinthoides, Asparagaceae [Hyacinthaceae]. Taxon.

[3] Raheem D, Thoss V (2016). Seasonal variation of mono-, di- and polysaccharides in British Bluebells (*Hyacinthoides non-scripta*). J. Plant Chem. Ecophysiol..

[4] Watson AA (1997). Glycosidase-inhibiting pyrrolidine alkaloids from *Hyacinthoides non-scripta*. Phytochem..

[5] Kato A (1999). Polyhydroxylated pyrrolidine and pyrrolizidine alkaloids from *Hyacinthoides non-scripta* and *Scilla campanulata*. Carbohydr. Res..

[6] Thoss V, Murphy PJ, Marriott R, Wilson T (2012). Triacylglycerol composition of British bluebell (*Hyacinthoides non-scripta*) seed oil. RSC Advances.

[7] Goto T, Kondo T, Tamura H, Takase S (1983). Structure of malonylawobanin, the real anthocyanin present in blue-colored flower petals of *Commelina communis*. Tetrahedron Letters.

[8] Takeda K, Harborne JB, Self R (1986). Identification of malonated anthocyanins in the Liliaceae and Labiatae. Phytochem..

[9] Mulholland DA, Schwikkard SL, Crouch NR (2013). The chemistry and biological activity of the Hyacinthaceae. Nat. Prod. Rep..

[10] Croft SL, Barrett MP, Urbina JA (2005). Chemotherapy of trypanosomiases and leishmaniasis. Trends Parasitol..

[11] Barrett, M. P. The rise and fall of sleeping sickness. *The Lancet* **367**, 1377–1378, 10.1016/S0140-6736(06)68591-7. 10.1016/S0140-6736(06)68591-7 16650633

[12] Hoet S (2007). Antitrypanosomal activity of triterpenoids and sterols from the leaves of *Strychnos spinosa* and related compounds. J. Nat. Prod..

[13] Ibrahim MA, Mohammed A, Isah MB, Aliyu AB (2014). Anti-trypanosomal activity of African medicinal plants: A review update. J. Ethnopharmacol..

[14] Lawal B (2015). Potential antimalarials from African natural products: A review. Int. J. Biochem. Re.s & Rev..

[15] Rasoanaivo P, Wright CW, Willcox ML, Gilbert B (2011). Whole plant extracts versus single compounds for the treatment of malaria: synergy and positive interactions. Mal. J..

[16] Dettmer K, Aronov PA, Hammock BD (2007). Mass spectrometry-based metabolomics. Mass Spectrom. Rev..

[17] Roessner U, Bowne J (2009). What is metabolomics all about?. Biotechniques.

[18] Yuliana ND, Khatib A, Choi YH, Verpoorte R (2011). Metabolomics for bioactivity assessment of natural products. Phytother. Res..

[19] Kamal N, Viegelmann CV, Clements CJ, Edrada-Ebel R (2017). Metabolomics-guided isolation of anti-trypanosomal metabolites from the endophytic fungus *Lasiodiplodia theobromae*. Planta Med..

[20] Tawfike, A. F. *et al*. Metabolomic tools to assess the chemistry and bioactivity of endophytic *Aspergillus* strain. *Chem Biodivers*. **14**, 10.1002/cbdv.201700040 (2017). 10.1002/cbdv.201700040 28672096

[21] Cheng C (2015). Biodiversity, anti-Trypanosomal activity screening, and metabolomic profiling of Actinomycetes isolated from Mediterranean sponges. PLoS One.

[22] Tawfike, A. F., Viegelmann, C. & Edrada-Ebel, R. In *Metabolomics tools for natural product discovery: methods and protocols* (eds Roessner, U. & Dias, A. D.) 227–244 (Humana Press, 2013).

[23] Roessner, U. & Beckles, D. In *Plant metabolic networks* (ed. Schwender J.) 39–69 (Springer, 2009).

[24] Villas-Boas, S. G., Nielsen, J., Smedsgaard, J., Hansen, M. A. E. & Roessner-Tunali, U. *Metabolome analysis: an introduction*., 24 (John Wiley & Sons, 2007).

[25] Macintyre L (2014). Metabolomic tools for secondary metabolite discovery from marine microbial symbionts. Mar. Drugs.

[26] Abdelmohsen UR (2014). Dereplication strategies for targeted isolation of new antitrypanosomal actinosporins A and B from a marine sponge associated-Actinokineospora sp. EG49. Mar Drugs.

[27] Worley B, Powers R (2013). Multivariate Analysis in Metabolomics. Curr. Metabolomics.

[28] Kettaneh N, Berglund A, Wold S (2005). PCA and PLS with very large data sets. Comput. Stat. Data Anal..

[29] Wold S, Sjöström M, Eriksson L (2001). PLS-regression: a basic tool of chemometrics. Chemom. Intell Lab Syst..

[30] Yang JY (2013). Molecular networking as a dereplication strategy. J. Nat. Prod..

[31] Wolfender J-L, Marti G, Thomas A, Bertrand S (2015). Current approaches and challenges for the metabolite profiling of complex natural extracts. J. Chromatog. A.

[32] Kind T, Fiehn O (2007). Seven golden rules for heuristic filtering of molecular formulas obtained by accurate mass spectrometry. BMC Bioinformatics.

[33] Buckingham, J. In *Dictionary of natural products on DVD* (ed. Buckingham J.) (Chapman and Hall/CRC, London, UK, 2015).

[34] Huber W, Koella JC (1993). A comparison of three methods of estimating EC50 in studies of drug resistance of malaria parasites. Acta Trop..

[35] Eriksson, L., Johansson, E., Kettaneh-Wold, N. & Wold, S. *Multi and megavariate data analysis*. (Umetrics AB 2006).

[36] Ebuele VO, Santoro A, Thoss V (2016). Phosphorus speciation by 31P NMR spectroscopy in bracken (*Pteridium aquilinum* (L.) Kuhn) and bluebell (*Hyacinthoides non-scripta* (L.) Chouard ex Rothm.) dominated semi-natural upland soil. Sci. Total Environ..

[37] Ori K, Koroda M, Mimaki Y, Sakagami H, Sashida Y (2003). Lanosterol and tetranorlanosterol glycosides from the bulbs of *Muscari paradoxum*. Phytochem..

[38] Crotti AEM (2004). The fragmentation mechanism of five-membered lactones by electrospray ionisation tandem mass spectrometry. Int. J. Mass Spectrom..

[39] Friedland SS, Lane GH, Longman RT, Train KE, O’Neal MJ (1959). Mass spectra of steroids. Anal. Chem..

[40] Sholichin M, Miyahara K, Kawasaki T (1985). Oligoglycosides of spirocyclic nortriterpenoids related to eucisterol. Chem. Pharm. Bull..

[41] Lee S-M (2002). Eucosterol oligoglycosides isolated from *Scilla scilloides* and their anti-tumor activity. Chem. Pharm. Bull..

[42] Ramos-Morales, E. *et al*. Improving the antiprotozoal effect of saponins in the rumen by combination with glycosidase inhibiting iminosugars or by modification of their chemical structure. *PLoS ONE* **12**(9) 10.1371/journal.pone.0184517. 10.1371/journal.pone.0184517 PMC5590940 28886130

[43] Watson DG (2013). A rough guide to metabolite identification using high resolution liquid chromatography mass spectrometry in metabolomic profiling in metazoans. Comput. Struct. Biotechnol. J..

[44] Nishida Y (2008). A new homostilbene and two new homoisoflavones from the bulbs of *Scilla scilloides*. Chem. Pharm. Bull..

[45] Adinolfi M (1987). Glycosides from *Muscari comosum*. 7. Structure of three novel muscarosides. Can. J. Chem..

[46] Parrilli M, Lanzetta R, Adinolfi M, Mangoni L (1980). Glycosides from *Muscari comosum*—III: The structure of further authentic aglycones. Tetrahedron.

[47] Kim J-W (2013). Triterpenoid saponins isolated from *Platycodon grandiflorum* inhibit hepatitis C virus replication. J. Evid. Based Complementary Altern. Med..

[48] Liu DL, Zhang X, Wang SP, Wang NL, Yao XS (2011). A new triterpenoid saponin from the roots of *Ardisia crenata*. Chin. Chem. Lett..

[49] Datta S (2014). Toxins in botanical dietary supplements: blue cohosh components disrupt cellular respiration and mitochondrial membrane potential. J. Nat. Prod..

[50] Zhou K (2009). Triterpenoids and flavonoids from celery (*Apium graveolens*). Journal of Natural Products.

[51] Zha L-y (2011). Anti-inflammatory effect of soyasaponins through suppressing nitric oxide production in LPS-stimulated RAW 264.7 cells by attenuation of NF-κB-mediated nitric oxide synthase expression. Bioorg. Med. Chem. Lett..

[52] Kim YS (2005). Isolation of a new saponin and cytotoxic effect of saponins from the root of *Platycodon grandiflorum* on human tumor cell lines. Planta Med.

[53] Choi YH (2010). Antiproliferative effects of saponins from the roots of *Platycodon grandiflorum* on cultured human tumor cells. J. Nat. Prod..

