# Supplementary material for: Application of metabolomics and molecular networking in investigating the chemical profile and antitrypanosomal activity of British bluebells (Hyacinthoides non-scripta)
Source: Sci Rep. 2019 Feb 22;9:2547. doi: 10.1038/s41598-019-38940-w (PMC6385288; doi:10.1038/s41598-019-38940-w)
Supplement: Supplementary file 1 — Supplemenary Information [file 41598_2019_38940_MOESM1_ESM.docx]

**Supporting Information for:**

**Application of metabolomics and molecular networking in investigating the chemical profile and antitrypanosomal activity of British bluebells (*Hyacinthoides non-scripta*)**

Dotsha J. Raheem^1¶#a^, Ahmed F. Tawfike^1, 2, 4¶#b*^, Usama R. Abdelmohsen^3^**^#c^**, RuAngelie Edrada-Ebel^2*^ and Vera Fitzsimmons-Thoss^1*^

^1^ School of Chemistry, Bangor University, Bangor, Gwynedd, UK.

^2^ Strathclyde Institute of Pharmacy and Biomedical Sciences, University of Strathclyde,

Glasgow, UK

^3^ Department of Botany II, Julius-von-Sachs Institute for Biological Sciences,

University of Würzburg, Germany.

^4^ Permanent address: Department of Pharmacognosy, Faculty of Pharmacy, Helwan University, Cairo, Egypt.

**^#a^ Current address:** Department of Chemistry, College of Science, University of Salahaddin, Erbil, Kurdistan, Iraq.

**^#b^ Current address:** Computational and Analytical Science Department, Rothamsted Research, Centenary building, Harpenden, Hertfordshire, UK.

**^#c^** **Current address:** Department of Pharmacognosy, Faculty of Pharmacy, Minia University, Minia 61519, Egypt

**^*^** Corresponding authors

E-mail: [vera.thoss@bangor.ac.uk](mailto:vera.thoss@bangor.ac.uk) (VFT)

**Sections:**

**Section1: Molecular networking and MS-MS application for the tentative identification of saponin aglycones**

**Section 2: Structural elucidation of bluebell flower saponin**

**Section 3: Ecological interpretation of relationships between plant parts, growth period and anti-trypanosomal activity**

**Section1: Molecular networking and MS-MS application for the tentative identification of saponin aglycones**

Molecular networking is a technique that allows investigating relationships between different metabolites obtained from the samples and thus connecting compounds with similar molecular mass peaks and / or fragmentation patterns to each other. The individual molecular mass peak in the network can be connected based on their fragmentations and the more similar, the more connected the network is. Fig S1 shows the complete molecular network for all metabolites in the bluebell extracts. The network was formed from all samples (bulbs, leaves, scapes and flowers) and showed the appearance of the metabolites colour coded to the tissue they were found in. This section focusses on the circled section in Fig 1 in order to tentatively probe the structure of the two related metabolites with [M-H]^-^ 469.26 and 455.28 which were identified to discriminate the bulb samples from the aerial parts (Fig 1, main text)

**Fig S1**. Molecular network of MS/MS data for all metabolites in Bluebell extracts. Each small circle representing a metabolite, colour coded according to their presence in one or more of the four groups G1: Bulbs G2: Leaves G3: Scapes G4: Flowers
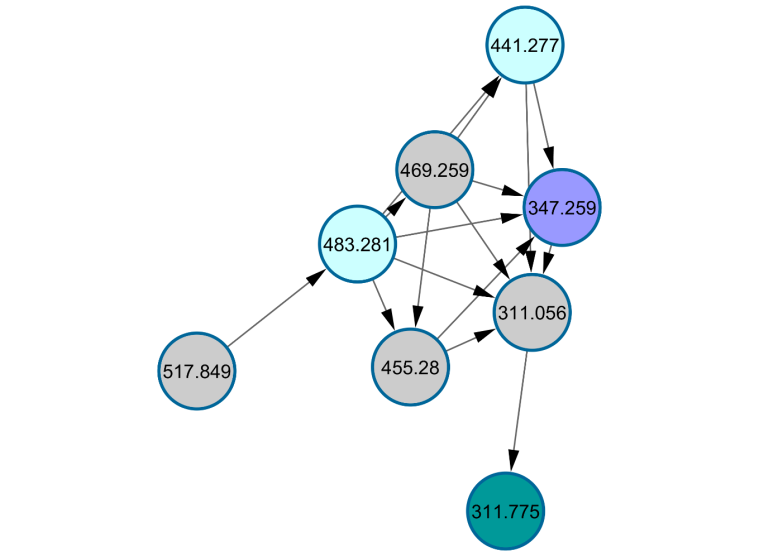
.
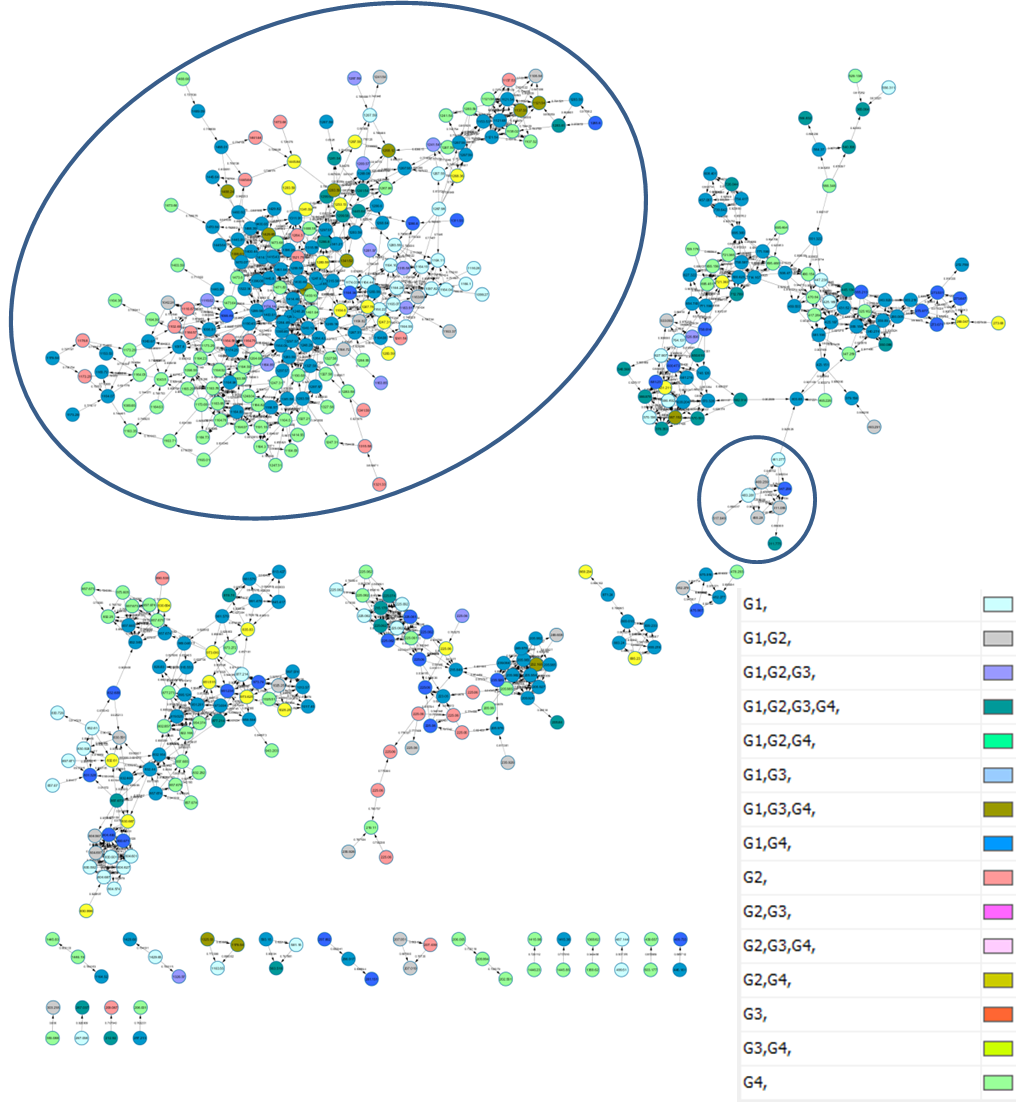


Based on the aglycone masses, the use of the DNP and mass fragmentation analysis, the structures in Fig S2 were suggested as hypothetical congeners.

**Fig S2**. Predicted structures of saponin aglycones found in bulb parts of bluebells based on their molecular ion peaks, ms/ms fragmentation data (Fig S3 and Fig 2) and comparison to hits found from the Dictionary of Natural Products particularly that of the aglycone of lucilianoside D from *Muscari paradoxum*  All hypothetical structures have a common mass ion peak fragment at *m/z* 347.2592,

Fig S3 shows the MS-MS fragmentation of the two aglycones matching the targeted molecular ion peaks of *m/z* 455.281 and 469.259 [M-H]^-^. The structures were acetylated congeners of the aglycone of lucilianoside D isolated from the fresh bulbs of the Japanese *Muscari paradoxum.* The fragmentation pattern for both ion peaks involved the subsequent loss of a keto group and a CO_2_ ion typical for a five-membered lactone ring system. A five-membered lactone ring fragmentation occurred simultaneously with the elimination H_2_O prior to the loss of a CO unit as observed in both of the spectra for *m/z* 455.281 and 469.259 [M-H]^-^. Further work is required to identify the structures of these two aglycones, however, the molecular networking approach provided useful guidance for this.

**Fig S3**: MS-MS traces for the metabolites with m/z 469.26 (A) and 455.28 (B) taken in negative mode and suggested loss of fragments on secondary and tertiary ionisation.

**Section 2: Structural elucidation of the bluebell flower saponin:**

**3*β*-(*O*-*β*-D-glucopyranosyl-(1→3)-*O*- *β*-D-glucopyranosyl-(1→3)-[*α*-L-rhamnopyranosyl-(1→2)]-*O*-*β*-D-glucopyranosyl-(1→2)-*O*-*α*-L-arabinopyranosyl-(1→6)-*O*-*β*-D-glucopyranosyl)oxy-17,23-epoxy-28,29-dihydroxy-27-norlanost-8-en-24-one**

^1^H NMR spectrum of this compound (Fig S7) showed a large number of signals distributed mainly on two regions of the spectrum: the signals from 0.75 – 2.65 ppm belonging to the aglycone and signals from 3.5 – 6.5 ppm attributed to the glycosidic part. The 2D correlation of these proton shifts with the relevant carbon signals in HSQC experiment (Fig S9) made the distribution of these groups even clearer. In the first region, a number of chemical shifts were readily identifiable as CH_3_-group signals by their relatively high intensity, multiplicities and orientation in DEPTQ spectrum (Figs S8 and S10). These included the singlet signals of C-18, 19 and 30, a doublet for C-21 and a triplet for C-26. Although the rest of the signals in this region were more overlapped in ^1^H NMR spectrum, their separation in DEPTQ and consequently in HSQC spectra allowed their identification. The arrangement of atoms and their linkages in the aglycone were identified combining the information from COSY, HMBC, and HSQC-TOCSY tests (Figs S11, S12 and S13). Table S1 lists ^1^H and ^13^C NMR data and Fig S and S5 show a schematic summary of 2D correlations utilised in the structural elucidation drawn based on the spectra (Figs S7, S9 to S13).

The proton on C-3 of the aglycone was found to be in the α-orientation based on the doublet of doublets signal with *J* value of 4.6, 11.8 Hz. Additionally, the NOESY spectrum (Fig S13) showed correlations between H-1*α*, H-2*α* and H-3. Since the methyl groups at C-10 and C-13 are usually in the *β*-orientation and C-14 is in the *α*-orientation, NOESY correlations with these groups were used in confirming and determining the spatial orientation of the other protons and/or methyl groups (Fig S5). Starting from the methyl group at C-10, correlations were found with H-2*β*, H-11*β* as well as the protons at C-28 indicating *β*-orientation of this group. H-5 was assigned the *α*-orientation because of its correlation with H-3*α*. The opposite orientations of C-5 and C-10 indicate a *trans* A/B ring junction. The methyl group at C-13 showed correlations with H-11*β*, H-15*β* and H-16*β*, while the methyl group at C-14 was correlated to H-7*α*, H-15*α* and H-16*α* indicating an opposite spatial orientation of the C-13 and C-14 methyl groups and therefore a *trans* C/D ring junction. A correlation between H-16*β* and H-20 indicated that the latter was in *β*-orientation. A similar correlation was also found between H-16*β* and H-22*β*, but not with H-23 which was correlated to H-22*α* and the methyl group at C-20 instead indicating that they were at α-orientation. Based on the biosynthesis and previous structural elucidation reports of saponins from the Hyacinthaceae, the results were consistent with the 17*S*, 20*R* and 23*S* configurations of the steroidal aglycone (1–3).

The presence of six sugars could be identified from their anomeric signals count in HSQC analysis (Fig S10). In most cases, except when the proton shifts are well resolved, *J* values were calculated from 2D cross peak. Accordingly, four of the anomeric protons showed *J* vales of 6.9 – 8.2 Hz were later identified as glucose. Arabinose with a small doublet of 3.1 Hz and finally rhamnose being the most downfield signal with a broad singlet proton (br s) signal. Therefore, the anomeric configurations were identified as *β* for the glucose units and *α* for arabinose and rhamnose.

**Fig S4**. NMR correlations obtained from COSY, HMBC and HSQC-TOCSY experiments used in the structural identification of DR5.

**Fig S5.** NOESY correlations of the aglycone part of the isolated steroidal saponin.

Starting from the anomeric protons, the neighbouring protons were identified using the COSY experiment (Fig S9). Protons and carbons belonging to the same sugar where identified from HSQC-TOCSY (Fig S12) and confirmed by HMBC data (Fig S11). The higher ^13^C chemical shifts of the C atoms involved in *O*-glycosidic bond formation helped in the initial identification of the interglycosidic linkages (points of branching). This involved C-6 of glucose 1, C-2 of arabinose, C-2 of glucose 2 and C-3 of both glucose 2 and glucose 3.

Arabinose was identified as a six-membered pyranose rather than a five-membered furanose ring conformer. This was based on the presence of HMBC correlations (Fig S11) between the anomeric CH and the CH_2_ groups; such correlation indicates the proximity of these two groups and would not have existed in a furanose ring. Also the ^13^C chemical shift values were in agreement with arabinopyranose rather than arabinofuranose as reported in (4) where the latter showed higher ^13^C values typical to furanose rings in particular with C-4 being higher than 80 ppm.

Some of the common sugars found in saponins are D-glucose, D-galactose, L-arabinose, L-rhamnose, D-xylose and D-glucuronic and D-galacturonic acids in addition to other less common one. The fact that the opposite enantiomers of these sugars are not detected in plants has been taken as a clue in identifying their configurations (5). Arabinose however, is an exception since it can occur in both D- and L- isomers in plants (6). Distinguishing the D- and L-arabinopyranose isomers has been achieved using ^1^H and ^13^C NMR analysis as the different isomers show different ^13^C chemical shifts and coupling constants of the anomeric protons (7). Chemical shifts and coupling values of the arabinose moiety in the isolated saponin showed closeness to the L- isomers reported in the paper rather than the D-isomer. Additionally, saponins from members of the *Hyacinthoideae* subfamily reviewed by (8) usually contain the same arrangement of the 3 - 4 first sugars in the oligosaccharide chain including L-arabinose. Based on the above, the identified sugars were assigned as D-glucose, L-rhamnose and L-arabinose. It has been indicated that *O*-glycosylation at C-2 of L-arabinopyranose favours the ^1^C_4_ conformation to reduce steric effect caused by the substituent (7).

**Table S1**. ^1^H and ^13^C NMR data for the isolated saponin in pyridine-D_5_. *J* values are calculated from 2D NMR cross peaks. Numbering of the aglycone moiety according to Fig 5.

| Position | δ_H_ ppm (multiplicity, *J* Hz) | δ_C_ ppm |
| --- | --- | --- |
|  |  |  |
| Aglycone |  |  |
| 1α  1β | 1.27 (*t-*like, 12.7)  1.75 (*d-*like) | 35.8 |
| 2α  2β | 2.39 (*m*)  2.13 (*dd*, 5.0, 13.0) | 27.3 |
| 3α | 4.50 (*dd*, 4.6, 11.8) | 82.1 |
| 4 | - | 48.2 |
| 5 | 2.19 (*m*) | 43.5 |
| 6α  6β | 2.15 (*m*)  1.75 (*m*) | 18.7 |
| 7α  7β | 2.00 (*m*)  2.19 (*m*) | 26.6 |
| 8 | - | 135.5 |
| 9 | - | 134.9 |
| 10 | - | 36.9 |
| 11α  11β | 2.19 (*m*)  2.00 (*m*) | 21.2 |
| 12α  12β | 1.45 (*m*)  2.43 (*m*) | 25.4 |
| 13 | - | 48.9 |
| 14 | - | 50.9 |
| 15α  15β | 1.33 (*m*)  1.65 (*m*) | 32.2 |
| 16α  16β | 2.16 (*m*)  1.59 (*m*) | 39.8 |
| 17 | - | 97.1 |
| 18 | 0.92 (*s*) | 19.4 |
| 19 | 1.05 (*s*) | 19.7 |
| 20 | 2.03 (*m*) | 43.8 |
| 21 | 1.02 (*d*, 7.2) | 17.3 |
| 22 | 1.76 (*m*), 1.98 (*m*) | 36.7 |
| 23 | 4.64 (*t*, 8.5) | 81.6 |
| 24 | - | 212.6 |
| 25 | 2.54 (*dq*, 2.9, 7.2) | 32.4 |
| 26 | 1.03 (*t*, 7.1) | 7.7 |
| 28a  28b | 3.90 (*m*)  4.62 (*d*, 12.5) | 62.7 |
| 29a  29b | 4.37 (*d*, 12.0)  4.93 (*d*, 12.9) | 61.0 |
| 30 | 1.47 (*s*) | 26.3 |
|  |  |  |
| D-Glucose 1 |  |  |
| 1 | 5.13 (*d*, 8.0) | 105.5 |
| 2 | 3.99 (*t*, 8.6) | 75.4 |
| 3 | 4.05 (*t*, 10.0) | 78.2 |
| 4 | 4.16 (*t*, 10.0) | 72.7 |
| 5 | 3.78 (*m*) | 75.4 |
| 6 | 4.17 (*m*), 4.44 (*dd*, 3.0, 12.0) | 68.6 |
|  |  |  |
| L-Arabinose |  |  |
| 1 | 5.27 (*d*, 3.1) | 101.0 |
| 2 | 4.65 (*m*) | 77.8 |
| 3 | 4.66 (*m*) | 71.6 |
| 4 | 4.55 (*m*) | 66.7 |
| 5 | 3.93 (*m*), 4.35 (*m*) | 62.7 |
|  |  |  |
| D-Glucose 2 |  |  |
| 1 | 5.14 (*d*, 6.9) | 102.5 |
| 2 | 4.18 (*t*, 8.1) | 76.7 |
| 3 | 4.06 (*t*, 11.2) | 89.3 |
| 4 | 4.10 (*t*, 10.0) | 69.2 |
| 5 | 3.58 (*m*) | 77.6 |
| 6 | 4.11 (*m*), 4.20 (*m*) | 61.8 |
| L-Rhamnose |  |  |
| 1 | 6.25 (*br s*) | 102.1 |
| 2 | 4.80 (*dd,* 1.9, 4.0) | 72.2 |
| 3 | 4.63 (*dd*, 5.1, 10.3) | 72.6 |
| 4 | 4.28 (*t*, 9.5) | 74.2 |
| 5 | 4.89 (*d,* 9.2) | 69.8 |
| 6 | 1.75 (*d*, 7.1) | 18.8 |
|  |  |  |
| D-Glucose 3 |  |  |
| 1 | 5.01 (*d*, 7.19) | 103.9 |
| 2 | 3.98 (*t*, 9.5) | 73.7 |
| 3 | 4.16 (*t*, 10.0) | 88.1 |
| 4 | 3.95 (*t*, 10.0) | 69.7 |
| 5 | 3.93 (*m*) | 78.0 |
| 6 | 4.14 (*m*), 4.44 (*dd*, 3.3, 10.5) | 62.1 |
|  |  |  |
| D-Glucose 4 |  |  |
| 1 | 5.19 (*d*, 8.2) | 105.3 |
| 2 | 4.06 (*t*, 9.3) | 75.2 |
| 3 | 4.19 (*t*, 9.1) | 78.2 |
| 4 | 4.15 (*t*, 10.0) | 71.6 |
| 5 | 3.97 (m) | 78.6 |
| 6 | 4.28 (*dd*, 6.0, 12.0), 4.52 (*dd*, 3.0, 12.0) | 62.4 |
|  |  |  |

The molecular formula C_64_H_103_O_34_ established from NMR spectroscopy was confirmed with HR-ESI-MS. The negative mode ionisation (Fig S14) showed a formate adduct of the molecular mass 1445.645 and a low intensity peak of [M-H]^-^ at *m/z* 1399.64. MS/MS analysis in this mode confirmed the mass of the oligosaccharide part which by deduction from the molecular ion gave the mass of the aglycone. On the other hand, the positive-mode ionisation gave valuable information about the number, sequence and types of the sugars present in the oligomeric chain (Fig S15). Fig S6 shows the main mass spectral fragments of the flower saponin in this mode. Fragments were found at *m/z* 447 and 418 corresponding to the loss of ethyl and propanoyl fragment from the aglycone. **Table S2** summarises the mass fragments obtained in both modes.

**Fig S6**. ESI-MS positive mode spectral fragments of the isolated saponin.

**Table S2**: ESI-MS/MS fragments for the isolated saponin in negative and positive fragmentation mode.

|  | ***m/z*** |
| --- | --- |
| **Negative mode fragments** |  |
| Molecular ion [M - H]^-^ | 1399.64 |
| Molecular ion + formate | 1445.64 |
| The glycan part + formate | 973.70 |
| **Positive mode fragments** |  |
| Molecular ion [M+H]^+^ | 1401.64 |
| [M - glucose + H]^+^ | 1239.59 |
| [M - rhamnose + H]^+^ | 1255.59 |
| [M – glucose - rhamnose + H]^+^ | 1093.54 |
| [M – (2 x glucose) + H]^+^ | 1077.54 |
| [M – (2 x glucose) - rhamnose + H]^+^ | 931.48 |
| [M – (3 x glucose) - rhamnose + H]^+^ | 769.43 |
| [M – (3 x glucose) - rhamnose - arabinose + H]^+^ | 637.39 |
| The aglycone | 475.34 |

The new saponin exhibited remarkably high activity when tested against blood stream *Trypanosoma brucei brucei* at a rate of 20 μM resulting in 98.9% inhibition.

**Fig S7.** ^1^H NMR spectrum of DR5 in pyridine-D_5_.

**Fig S8.** DEPTQ spectrum of DR5 in pyridine-D_5_.

**Fig S9.** ^1^H-^1^H COSY spectrum of DR5 in pyridine-D_5_.

**Fig S10.** ^1^H-^13^C HSQC spectrum of DR5 in pyridine-D_5_.

**Fig S11.** ^1^H-^13^C HMBC spectrum of DR5 in pyridine-D_5_.

**Fig S12:** HSQC-TOCSY spectrum of DR5 in pyridine-D_5_

**Fig S13.** NOESY spectrum of DR5 in pyridine-D_5_

**Fig S14.** HR-ESI-MS of DR5 in negative mode


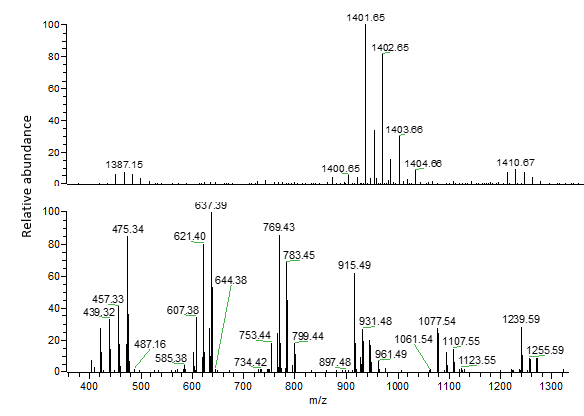


B

A

S 1 Fig. HR-ESI-MS (A) and MS/MS CID of [M+H]^+^ at *m/z* 1401.65 (B) of DR5 in positive mode

References:

1. Kuroda M, Mimaki Y, Ori K, Koshino H, Nukada T, Sakagami H, et al. Lucilianosides A and B, two novel tetranor-lanostane hexaglycosides from the bulbs of Chionodoxa luciliae. Tetrahedron. 2002;58(33):6735–40.

2. Kuroda M, Mimaki Y, Ori K, Sakagami H, Sashida Y. 27-Norlanostane Glycosides from the Bulbs of Muscari p aradoxum. J Nat Prod. 2004;67(12):2099–103.

3. Ori K, Kuroda M, Mimaki Y, Sakagami H, Sashida Y. Norlanostane and lanostane glycosides from the bulbs of Chionodoxa luciliae and their cytotoxic activity. Chem Pharm Bull. 2003;51(1):92–5.

4. Adinolfi M, Barone G, Lanzetta R, Laonigro G, Mangoni L, Parrilli M. Glycosides from Muscari comosum. 5. Structure of muscaroside B. Can J Chem. 1984;62(6):1223–6.

5. Massiot G, Lavaud C. Structural elucidation of saponins. Stud Nat Prod Chem. 1995;15:187.

6. Bhat S, Nagasampagi B, Sivakumar M. Carbohydrates. In: Bhat S, Nagasampagi B, Sivakumar M, editors. Chemistry of Natural Products. India: Narosa Publishing House; 2005. p. 458.

7. Ishii H, Kitagawa I, Matsushita K, Shirakawa K, Tori K, Tozyo T, et al. The configuration and conformation of the arabinose moiety in platycodins, saponins isolated from platycodongrandiflorum, and mi-saponins from madhucalongifolia based on carbon-13 and hydrogen-1 nmr spectroscopic evidence: The total structures of the sapo. Tetrahedron Lett. 1981;22(16):1529–32.

8. Mulholland DA, Schwikkard SL, Crouch NR. The chemistry and biological activity of the Hyacinthaceae. Nat Prod Rep. 2013;30(9):1165–210.

**Section 3: Ecological interpretation of relationships between plant parts, growth period and anti-trypanosomal activity**

Saponins are plant defence compounds. They are widely expressed in the plant kingdom and specific biological activities of distinct saponins have been documented. The synthesis of saponins and chemical alterations of the aglycone occurs in response to environmental factors. In addition, different plant parts have been shown to synthesise saponins (for further information see reviews by Augustin et al., 2011 and Moses et al., 2014). The samples used in this study were designed to also assess both the variations in metabolite profile during the annual growth phase of a perennial bulbous plant and to assess the chemical variation in different plant parts during this growth phase. As shown in Figure 1 in the text, the below-ground parts, the bulb, showed a distinctly different metabolic profile compared to the above ground parts, in particular containing fewer glycosides. However, the bulb showed clear differences in its metabolite profile for the four samples taken before flowering (24/3, 3/4, 16/4 and 1/5), forming one cluster (Fig 2, group 1) and after flowering during seed ripening (12/6 and 3/7) forming another cluster (Fig 2, group 2). The first period is characterised by the bulb supplying the growing plant with reserves contained within it. This results in the bulb losing a large proportion of its weight to the growing above-ground plant which culminates in the old bulb being shed and a new bulb being formed. The second cluster coincides with the relocation of metabolites from the senescing above ground parts into the new bulb. During the flowering period (15/5 and 29/5) photosynthate is in excess and relocated into the new bulb (3). The PCA score plot (Fig 1 main text) shows that the above-ground plant parts tend to show higher molecular weight. This is most likely to have resulted from glycosylation of metabolites which modulates stability, biological activity (as shown for the saponin in this study), solubility signalling for storage or intra- and intercellular transport (Augustin et al., 2011).

The biological activity elucidated from the antitrypanosomal assays strongly suggests the suggested saponins to be contributing to the effects recorded in the assays. The structurally elucidated saponin, which is a lanosterol glycoside, is uncommon (Moses et al., 2015), however, spirocyclic nortriterpernoids are reported from the *Hyacinthoideae* (Mulholland et al., 2013). It is unclear what proportion of the saponins are biosynthesised during each growth period versus being relocated between above and below-ground plant parts. Saponins have been reported to increase plant defense and are biosynthesised and modified in different plant parts. In addition, loss of saponins from the plant into the rhizosphere has also been documented (Augustin 2011, Moses 2014)

This study used methanol extracts as its basis. Methanol extracts include carbohydrates, phenolics, saponins and other either more polar or glycosylated metabolites. The proportion of carbohydrates in the extract affects its biological activity. Bluebell bulbs contain between 35 to 80 % non-structural carbohydrates (3), with the highest concentration occurring during peak flowering and the lowest before leaf emergence above ground. The cause for the inactivity of the bulb extracts during the below ground and early growth period, assuming that they might contain the saponins, is hypothesised to relate to the carbohydrate content diluting the concentration of saponin in the extract. There were only 2 active extracts (Table 2 main text): 16^th^ April which coincided with the period when the old bulb is being shed and the new bulb is being formed. In addition, this sample was taken during the most active growth phase and biosynthesis of saponins for plant protection is likely to be high. The second active bulb extract was taken on 29^th^ October when the carbohydrate content of the bulb is lowest before above ground emergence of the shoot (3). The shoot and flower samples were also highly active for the period when the old bulb is shed, suggesting that precious metabolites are contained within the plant and its contribution to soil organic matter via the old bulb is minimal (32,33).

Bluebell flowers are formed as part of the emerging shoot. Shoots were found active on 3^rd^, 16^th^ and 24^th^ March. Only from the next sampling occasion onwards (3^rd^ April until 17^th^ July) were flower samples separated from the scapes. The highest inhibition for flowers was recorded for the 3^rd^ and 16^th^ April. Assuming the saponin content to be responsible for the observed inhibition, both shoot and flower extracts indicate possible relocation from the bulb after glycosylation and *denovo* biosynthesis. Peak flowering, the period when for most individuals of the population the flowers are standing clearly upright, colour is at its maximum and pollination is taking place, occurred between 8^th^ to 29^th^ May. The flower sample taken during the peak flowering season showed lesser inhibition of *Trypanosoma brucei brucei*. Assuming that plant resources are required for attraction of pollinators, the reduction in inhibition could reflect a shift in allocation of resources to the biosynthesis of flower colour and nectar production versus saponin defense. Extracts of all other flower samples were highly active. After peak flowering seed capsules are formed and the seeds ripen therein.

All leave sample extracts were also highly active, showing the strongest inhibition for extracts obtained from leaves during peak flowering. Like the flowers, the leaves were equally included in the shoots for the previous sampling occasion, supporting the hypothesis that some saponins are relocated into the above ground plant parts during bulb renewal. Bluebells have only a small amount of biomass and no means to regrow leaves, if lost as the flower bud and the number of leaves are laid down in the newly formed bulb at the beginning of the annual growth cycle which makes there need for chemical defence more pressing.

References:

Augustin, J. M., Kuzina, V., Andersen, S. B. and Bak, S. (2011) Review: Molecular activities, biosynthesis and evolution of triterpenoid saponins. Phytochemistry 72: 435-457.

Moses, T., Papadopoulou, K.K. and Osbourne, A. (2014) Metabolic and functional diversity of saponins, biosynthetic intermediates and semi-synthetic derivatives. Critical Reviews in Biochemistry and Molecular Biology 49: 439-462.
